# Defining tropism and activity of natural and engineered extracellular vesicles

**DOI:** 10.3389/fimmu.2024.1363185

**Published:** 2024-04-10

**Authors:** Wooil Choi, Dong Jun Park, Brian P. Eliceiri

**Affiliations:** ^1^Department of Surgery, University of California San Diego, La Jolla, CA, United States; ^2^Department of Dermatology, University of California San Diego, La Jolla, CA, United States

**Keywords:** extracellular vesicles (EVs), EV tropism, EV surface engineering, EV ligands, targeting EV, adaptive immunity

## Abstract

Extracellular vesicles (EVs) have important roles as mediators of cell-to-cell communication, with physiological functions demonstrated in various *in vivo* models. Despite advances in our understanding of the biological function of EVs and their potential for use as therapeutics, there are limitations to the clinical approaches for which EVs would be effective. A primary determinant of the biodistribution of EVs is the profile of proteins and other factors on the surface of EVs that define the tropism of EVs *in vivo*. For example, proteins displayed on the surface of EVs can vary in composition by cell source of the EVs and the microenvironment into which EVs are delivered. In addition, interactions between EVs and recipient cells that determine uptake and endosomal escape in recipient cells affect overall systemic biodistribution. In this review, we discuss the contribution of the EV donor cell and the role of the microenvironment in determining EV tropism and thereby determining the uptake and biological activity of EVs.

## Introduction

Extracellular vesicles (EVs) are observed in most biological fluids and are notable for their wide range of sizes, payloads, and biological activities. As defined by the MISEV 2023 guidelines, we focus here primarily on small EVs that are under 200 nm (1–5). EVs convey protein, lipid, and RNA payloads to surrounding or distant recipient cells (6–8). EV-mediated physiological processes affect not only recipient cells but also the surrounding microenvironment, depending on the heterogeneity of EVs and, consequently, regulate many physiological systems including the immune system (9, 10). EVs that are secreted by both non-immune cells and immune cells mediate immune stimulation or suppression. Tumor-derived EVs contain specific markers, which induce phenotypic changes in recipient cells. For example, EVs derived from immune cells, specifically antigen-presenting cells (APCs), carry cargos that can directly induce the peripheral immune response (11). Studies of EV-mediated immune regulation provide insights for therapeutic approaches to treat cancer (12, 13), aging (14) as well as inflammatory diseases (15, 16). These reports demonstrate the potential of EVs as physiologically relevant therapeutics that modulate recipient cells through luminal or transmembrane payload delivery.

This review focuses on the specific challenges of engineering EVs to express essential surface molecules to direct tropism. There have been several excellent recent reviews that provide details on EVs as therapeutics in wound healing (17). EVs are naturally occurring nanoparticles that are generally biocompatible and efficient therapeutics in various systems that provide a foundation to design and deliver novel cargoes for next-generation therapeutics. This review addresses the importance of engineering tropism into the design of EVs that are relevant for biodistribution that we argue is important because there few clinical studies with engineered EVs.

Along with the potential for EVs to deliver therapeutic cargo, several engineering strategies have been reported for luminal loading or surface display of functional proteins or RNA. EVs can be engineered with known or screened cargo and enhance the therapeutic efficacy in disease models (18–21). Here, we review cell and organ tropism of EVs depending on the source and cellular target, and engineering strategies for EV surface display for targeting delivery.

## Understanding EV tropism based on cellular source and relevance to extracellular microenvironment

In addition to differences in EV payloads, which include proteins (i.e., enzymes, cytokines, growth factors, and their receptors) and nucleic acids (mRNA, miRNA, and DNAs), the lipid composition of EVs is determined by the cell source (22). While many studies identify novel proteins that mediate tropism, existing datasets like those available from ExoCarta (ExoCarta.org) can be mined to establish relevance from various tissues, cell types, and experimental models. These molecular signatures provide insight into the interactions of EVs with target cells and its biological activity. For example, EVs from different cell sources produce specific transcriptional responses in recipient cells. Specifically, mesenchymal stem cell (MSC)–derived EVs induce wound healing, epithelial cell–derived EVs up-regulate cell adhesion, immune cell–derived EVs activate inflammatory genes, and most EVs associate with recipient fibroblasts (18). EV composition is determined largely by the type and physiological state of the producer cells (19). EVs show a similar lipid profile as the cell that produced them, including glycosphingolipid, phospholipid, and cholesterol composition (20, 21, 23). Proteins displayed on the surface of nearly all EVs include Alix, tetraspanins (CD9, CD63, and CD81), heat shock protein 70 (24, 25), and other proteins that are often associated with specific EV subsets (24, 26, 27).

Protein interactions at the interface between EVs and recipient cells provide the basis for EV uptake and tropism. For example, enzymatic depletion of heparan sulfate proteoglycans (HSPGs) on the cell surface or inhibition of endogenous proteoglycan synthesis attenuated uptake of cancer cell–derived EVs (28). These reports show that EV uptake is mediated by protein-protein interactions between EVs and recipient cells, including immune cells (29, 30). Digestion of surface protein by proteinase K shows significantly decreased EV uptake (31–33) providing examples of how surface proteins mediate interactions with recipient cells.

**Figure 1:**
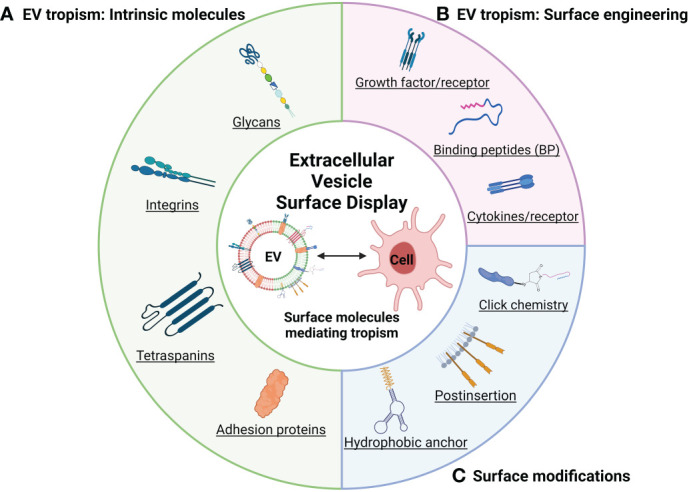
Schematic illustration of representative EV tropism and modifications based on surface display (created with BioRender.com). **(A)** Surface display depending on the cell source determines EVs tropism. **(B)** Surface display targeting EV tropism by endogenous expression. **(C)** Summary of engineering methods for EV surface modification.

Integrin and tetraspanin proteins, canonical components of EVs, mediate the interaction of the EV with recipient cell membranes (**Figure 1A** and **Table 1**). Alpha and beta subunits in the heterodimeric integrin complex determine the specificity for binding to the extracellular matrix (45, 46). There are over 18 alpha-subunit variants and eight beta-subunit variants in the integrin family. Circulating platelet–derived EVs interact with integrin α_M_ (CD11b) and Tyro3, Axl, and Mer (TAM) tyrosine kinase receptors on the monocytes and granulocytes in a Ca^2+^-dependent manner (34). Exosomal integrin α_v_β_6_ can regulate the M2 inflammatory response of CD14^+^ monocytes (35). Colorectal cancer cells release integrin β-like 1-rich EVs to the circulation in the blood stream to activate resident fibroblasts in remote organs. These activated fibroblasts induce formation of the pre-metastatic niche and promote metastasis by secreting pro-inflammatory cytokines (36). Exosomal integrins α_6_β_4_, α_6_β_1_, and α_v_β_5_ bind lung fibroblasts, epithelial cells, and liver-resident macrophages, respectively, and promote inflammatory responses in recipient cells (37). Tropism of palmitic acid–stimulated hepatocyte-derived EVs to hepatic stellate cells is an example of a biochemical modification that promotes EV tropism based on increased expression of integrins α_v_β_3_ and α_8_β_1_ (47). EVs derived from bone-tropic breast cancer show a high expression of the integrin-binding sialoprotein. These stimulated EVs have tropism to osteoclasts and promote bone metastasis (38). The integrin α_v_-VAMP complex–associated protein A (VAPA) on hepatocellular carcinoma–derived EVs promotes bone metastasis. EV-mediated integrin–VAPA complexes are activated in the plasma membrane of recipient osteoclasts and result in actin cytoskeleton formation in osteoclasts (39).

**Table 1:** EV surface display, tropism, and target ligands according to literature.

EV source	Surface display	Tropism	Target ligands	Reference
Platelet	Integrin α_M_	Monocyte and granulocyte	TAM tyrosine kinase receptor	(34)
Prostate cancer cell	Integrin αVβ6	Monocyte	CD14	(35)
Colorectal cancer cell	Integrin β-like 1	Fibroblast	TNF-α–induced protein 3	(36)
Tumors	Integrin α_6_β_4_	Lung fibroblast	-	(37)
Tumors	Integrin α_6_β_1_	Lung epithelial cell	-	(37)
Tumors	Integrin α_v_β_5_	Liver-resident macrophage	-	(37)
Bone tropic breast cancer cell	Integrin-binding sialoprotein (IBSP)	Osteoclast cell in bone	IBSP receptor	(38)
Bone tropic hepatocellular carcinoma	Integrin α_v_	Osteoclast cell	VAMP complex–associated protein A	(39)
Pancreatic cancer cell	Tspan8–integrin α_4_	Pancreas-resident endothelial cell	CD54	(40)
Pancreatic cancer cell	CD49d and CD106	Aortic endothelial cell	-	(41)
Pancreatic cancer cell	CD151 and CD49c	Lung-resident fibroblast	-	(41)
Bone marrow dendritic cell	CD9, CD1, and MFG-E8/lactadherin	Spleen-resident dendritic cell	Integrin α_v_β_3_ and integrin α_L_	(42)
Cancer-associated stromal fibroblast cell	Annexin A6	Pancreatic tumor cell	CD9	(43)
Colorectal cancer cell	ADAM17	Colorectal cancer cell and peritoneal mesothelial cell	Integrin α_v_β_1_	(44)

Tetraspanins function as integral membrane proteins and are widely studied as canonical EV markers that also provide insights into how they may be biologically active. For example, tetraspanin 8 (Tspan8), a member of the tetraspanin family of proteins, induces cellular tropism by forming a complex with other tetraspanins (CD9 and CD81) or integrins (α_4_β_4_) on the EV membrane. EVs expressing the Tspan8–integrin α_4_ complex show selectivity for CD54 as a major ligand on endothelial and pancreatic cells (40). CD49d (integrin α_4_) and CD106 (vascular cell adhesion molecule 1) on EVs recruited by Tspan8 induce cellular uptake by aortic endothelial cells, and CD151 and CD49c (integrin α_3_) are implicated in EV uptake by lung-resident fibroblast (41). Uptake of EVs by spleen-resident dendritic cells (DCs) is mediated via tetraspanins (CD9 and CD81), milk fat globule (MFG)–E8/lactadherin, and phosphatidylserine on the EVs and integrin α_v_β_3_, integrin α_L_ (CD11a), and CD54 (intercellular adhesion molecule 1) on DCs (42). Suppressing CD9 action with function-blocking antibodies decreases EV uptake and cargo transfer to recipient by endocytosis (48). Cell surface CD9 also can mediate the aggressiveness of pancreatic tumor cell interactions with annexin A6^+^ (ANXA6+) EVs. These EVs are derived from cancer-associated stromal fibroblasts, which induce the MAPK pathway, cell migration, and epithelial-to-mesenchymal transition (43). In contrast, lack of CD9 expression on EVs produced by colorectal cancer cells enhanced interaction between integrin α_v_β_1_ and its ligand ADAM17 and uptake by recipient cells (44). These reports suggest that integrins, tetraspanins, and their ligands are involved in the tropism of EVs. In the context of understanding the functional role of tetraspanins in the biogenesis of EVs, several reports have shown that increased expression of tetraspanins increases the number of EVs formed (49), whereas knockdown of tetraspanins has led to minimal reductions in EV formation (50, 51). Therefore, more studies are required to understand the function of specific tetraspanins in inducible and cell-type–specific genetic systems to better define their role in intercellular signaling vs. EV biogenesis (52).

## Understanding EV tropism based on cellular targets

EVs have been demonstrated to improve cellular processes associated with wound healing, including proliferation, migration, and angiogenesis. Evidence of this progress is a phase I clinical trial of healthy volunteer adults that is currently underway to evaluate safety in the context of wound healing (53, 54). MSC-derived EVs have been shown to be involved in reduction of échelle d’évaluation clinique des cicatrices d’acné (ECCA) score, a measure of scarring, by about 12.6% more than a control group in a small sample set of 25 patients (53). Platelet-derived EVs show evidence for the safety and therapeutic utility (54). Several reports have focused on their role as growth factors in tissue repair and regeneration. Vascular endothelial growth factor A (VEGF-A), fibroblast growth factor 2 (FGF-2), hepatocyte growth factor, and platelet-derived growth factor are key growth factors that are known to promote wound healing in various animal models (55, 56). More recently, it has been proposed that EVs can deliver specific growth factors that may facilitate their biodistribution and overall pro-reparative activity (57). For example, it has been reported that EVs with growth factor payloads can improve tissue regeneration when displayed on the surface of EVs. FGF-2 bound EVs secreted from dermal fibroblasts activate the extracellular signal-regulated kinase (ERK) and signal transducers and activators of transcription (STAT) signaling pathways and enhance cell proliferation and migration in recipient cells (58). Endogenous expression of VEGF and transcription factor EB on EVs derived from endothelial cells improves vascularization, attenuates muscle injury, and improves recovery of limb function through VEGF/Vascular endothelial growth factor receptor (VEGFR) pathway and autophagy-lysosome pathway in critical limb ischemia mice model (59). In other studies, adipose tissue–derived EVs that overexpressed VEGF fused with CD63 on the surface showed increased tube formation, cell proliferation, and migration of lymphatic endothelial cells. Continuous EV treatment rescues lymphedema in a mouse model indicating controlled EV kinetics can be stable *in vivo* (60). Bone morphogenetic protein 2 displayed on EVs derived from bone marrow–derived MSC enhances osteogenic differentiation while maintaining the function of bone marrow MSC EVs (61). These studies are consistent with a role for growth factors on EVs in promoting tissue repair and regeneration and support the further refinement of the loading and display of specific growth factors targeting specific cell types relevant in injury and tissue repair (62).

In addition to the display and expression of known peptides like growth factors, phage display can be used for the discovery of novel peptides that are focused on naturally occurring peptides that can be displayed on EVs to target recipient cells. For example, expression of a fusion of the cardiomyocyte binding peptide with Lamp2b on EVs results in increased efficiency of EV uptake into cardiomyocytes and decreased cardiomyocyte apoptosis associated with higher cell retention (63). A fusion of the Lamp2b protein with the ischemic myocardium targeting peptide (CSTSMLKAC) expressed on MSC-derived EVs reduced fibrosis and enhanced vascular neogenesis, and cardiac function could be monitored in ischemic heart tissue (64). The therapeutic potential of EVs has been observed in skin repair (65) as well as in kidney (66). These studies show the promise in designing engineered EVs to produce a synergistic effect on wound healing and tissue regeneration by expression of growth factor EVs.

Expression of peptides on EVs has been explored as a mechanism to target EVs to tumor cells. For example, interleukin-3 (IL-3) expressed on the surface of EVs can be used to target the delivery of EVs to the IL-3 receptor that is highly expressed in chronic myelogenous leukemia cells compared with that in normal hematopoietic cells (67). High expression of EGFR on tumors of epithelial origin suggests that the EGF-binding peptide, nanobody, and aptamer (68–70) on the surface of EVs could be used to target the delivery of EVs for uptake by epithelial tumor cells. CD19 (71), CD20 (72), integrin (73), and other ligands are known to be highly expressed on tumor cells and could potentially be used to target EVs to these cells. In an alternative approach, EVs may be useful for modifying the immune microenvironment for cancer immunotherapy (74, 75). TNFR1 stimulation by tumor necrosis factor alpha (TNF-α)–decorated EVs can induce activation of necroptosis and cell death in cancer (76). Targeting the APCs via EVs provides specific antigens for cancer therapy. For example, APCs targeting EVs derived from tumor cells provide antigen to APCs cells, and it could enhance tumor recognition (77, 78). Reprogramming of immune cells by immune cell–targeting EVs shows the potential of EVs as a novel immunotherapeutic agent (79, 80). EVs derived from macrophages can modulate the immune microenvironment of tumors. Reprogramming of tumor-associated macrophages (TAMs) to a M1 macrophage via targeting TAMs or tumor cells by engineered M1-derived EVs decorating IL-4 receptor or CD47 via postinsertion of targeting peptide increased anti-tumor immunity (81, 82). These reports show the potential of cancer treatment through a direct tumor targeting strategy loaded with anti-cancer drug and control of the surrounding immune microenvironment through modification of EV surface display (**Figure 1B**).

## EV tropism is demonstrated by recent examples

EV-mediated cargo delivery and EV characteristics such as toxicity, immunogenicity, and engineerability are being tested in various diseases models as a cell-free therapy (83, 84). Exploring the feasibility of engineering the surface composition of EVs is important to enable cell or organ specific delivery and *in vivo* tracking (85, 86). Therapeutic approaches to treat cancer therapy based on cell source through engineered EV are also being explored (87). Several approaches to introduce targeting moieties are being applied directly to EVs or indirectly to cells (88).

In addition to the use of phage display to target cardiomyocytes, this approach can be used for various other cell types to screen for peptides that have cell-type–specific affinity. In the example of targeting ischemic cardiac tissue, phage display was performed with a fusion with Lamp2b protein on the surface of EVs (64). EVs engineered with silk fibroin patch (SFP)–biding peptides that were identified by phage display showed dose-dependent accumulation of the SFP and enhanced wound closured efficacy in the diabetic wound model (89). CD9 and CD63 are known as EVs markers, and CD63 is a well-known EV scaffold protein that is often used to endogenously express or decorate foreign peptide (29, 90). Endogenous display of target binding peptides via CD63 shows stable expression and specificity. For example, expression of glycosylation domains on the large extracellular loop of CD63 show specificity toward endothelial cells and DCs (62, 91). Screened peptides identified by phage display were decorated using CP05, which directly bind to the CD63 second loop (92). As another approach, transgenic mice expressing Eµ-myc were used to select for a Eµ-myc B lymphoma–specific phage sequence through several bio-panning cycles after intravenous injection. The malignant B cell–specific sequence recognizes cancer-derived exosomes (93). Together, several studies have shown the power of phage display to identify target-specific binding peptides that can be engineered onto EVs.

Direct modification of the EV surface (**Figure 1C**) using hydrophobic anchors, rather than endogenous expression, also exists. Cholesterol is also one component of EV membrane, which spontaneously inserts into the membrane via its hydrophobic moiety (94), and cholesterol-modified RNA structures can be displayed on the EV surface that improves the efficiency of delivery of the modified EVs (68). Similarly, ceramide and dioleoyl phosphatidylethanolamine (DOPE) are being explored for their lipid anchoring properties with DOPE showing the most efficient anchoring effect and stable retention (95).

Another approach to engineering EVs is to use functional moieties that can be engineered onto the surface of EVs that can then be functionalized by chemical reactions using a technology termed click reactions. Click reactions can introduce large molecules on the EV membrane, and a greater amount of quantitative decoration is possible than via endogenous expression (96). Over-modification of EV proteins with functional groups, which are necessary for click reactions, might inhibit their intrinsic surface properties (97). Another approach being explored uses a click reaction to attach a targeting moiety to polyethylene glycol (PEG), and the resulting PEG-anchored peptide inserted in the EVs to gives functionality (98). In this way, in addition to the properties of EVs such low toxicity, low immunogenicity, and functional cargo and surface proteins, EVs with added functionality through surface engineering are being tried in various disease models.

To address the translational progress of EV research to the clinic, we review here recent trials based on a literature search using the words “extracellular vesicles” in the ClinicalTrials.gov database. We reviewed a total of 81 registered studies and categorized them on the basis of the following: 1) biomarker and diagnosis accounted for 50 cases (62.5%); therapeutics-related studies accounted for 26 cases (32.1%) and 18 cases (22.2%) in phases 1 to 3 as summarized in **Table 2**. Various organs were the subject of these studies: lung (six cases), skin (three cases), gut (three cases), liver (two cases), heart (one case), eye (one case), ear (one case), and ovary (one case). Although EVs in clinical trials generally use unmodified MSC-derived EVs, the future of EV therapies will likely expand to include sources from other cell types and utilize engineered EVs (i.e., ones that enhance tropism and deliver therapeutic payloads).

**Table 2:** Clinical trials related to EVs as therapeutics (clinicaltrials.gov).

Disease	Phase	Administration	EV source	Clinical Trial ID
Burn wound	1	Topical	MSCs	NCT05078385
Acute respiratory distress syndrome (ARDS)	2	Intravenous	Bone Marrow (BM)	NCT04493242
ARDS	3	Intravenous	BM-MSCs	NCT05354141
ARDS	2	Intravenous	MSCs	NCT06002841
ARDS	2	Intravenous	BM-MSCs	NCT05127122
Drug-refractory left ventricular	1	Intravenous	Cardiovascular progenitor cells	NCT05774509
Retinitis pigmentosa	2	Intravitreal	BM-MSCs	NCT06242379
Mild-moderate COVID-19	2	Intravenous	BM-MSCs	NCT05125562
Chromic tympanic membrane perforation	3	Topical	Blood	NCT04761562
Epidermolysis bullosa	2	Topical	MSCs	NCT04173650
Acute-on-chronic liver failure after liver transplantation	1	Intravenous	MSCs	NCT05881668
Post–COVID-19 syndrome	2	Intravenous	BM-MSCs	NCT05116761
Acute-on-chronic liver failure	2	Intravenous	MSCs	NCT05940610
Premature ovarian failure	2	Intraovarian	MSCs	NCT06202547
Ulcerative colitis	1	Intravenous	MSCs	NCT05176366
Crohn’s disease	1	Intravenous	MSCs	NCT05130983
Perianal fistulas	1	Intravenous	MSCs	NCT05836883
Healthy volunteer	1	Topical	MSCs	NCT05523011

## Tropism based on an understanding of EV systemic biodistribution

**Figure 2:**
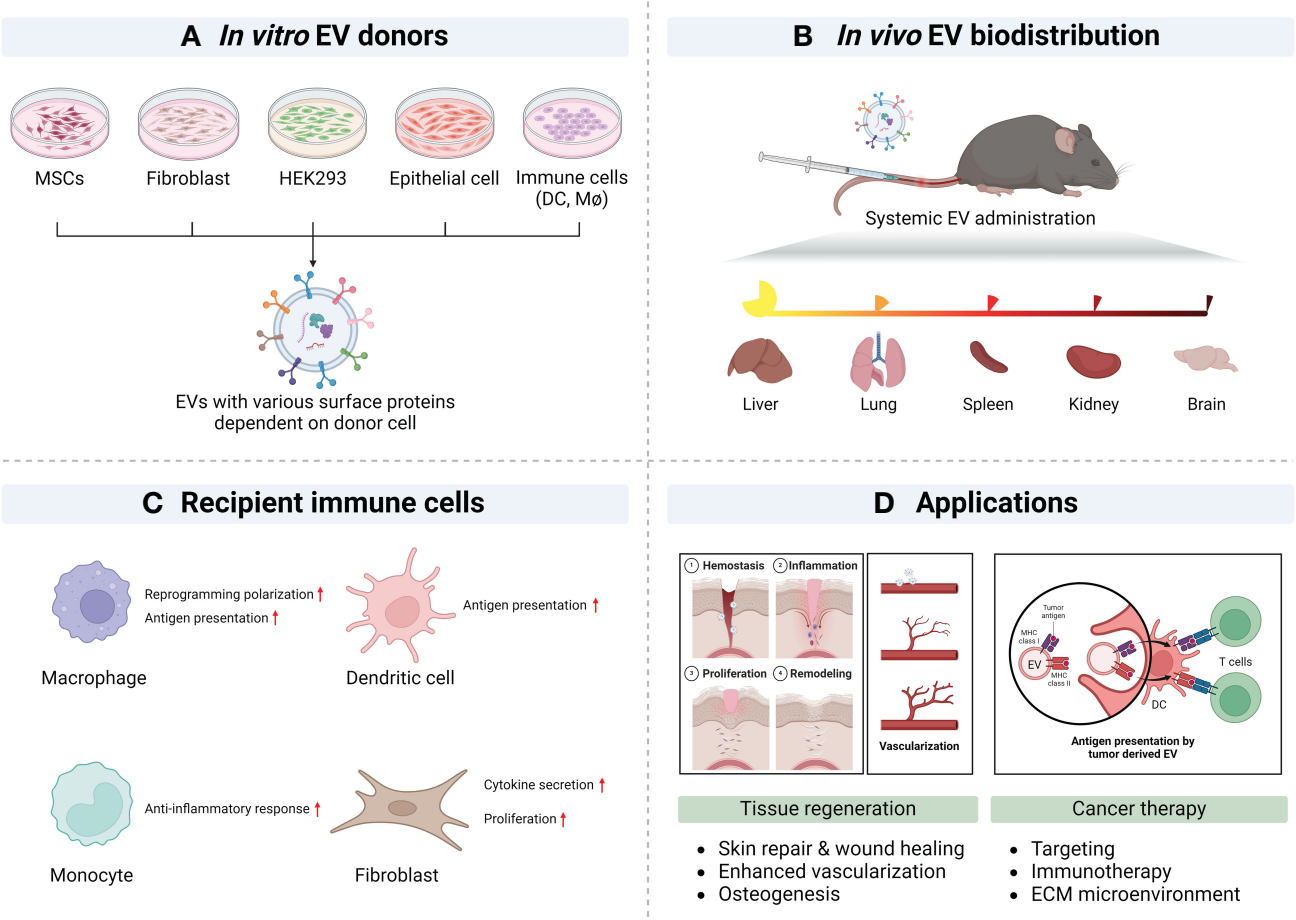
Natural tropism of EV and potential applications (created with BioRender.com). **(A)** Representative EV donor cells *in vitro*. **(B)** Summary of EV biodistribution studies in mouse models. **(C)** Representative EV’s immune modulation. **(D)** Summary of applications of surface modified EVs.

Classical EV tracking studies have relied on fluorescent membrane dyes, but several reports suggest that such labeling methods may negatively impact the sensitivity and/or not provide an accurate demonstration of the distribution in cells and tissues (99). Several tracers have been assessed to track the biodistribution of EVs (100), including their off-target distribution, which is important understand when assessing the therapeutic potential of EVs (**Figure 2A**) (101). mCherry red has been used for EV tracking *in vivo*, but its utility is limited by a poor signal to noise ratio. However, DiR [1,1′-dioctadecyl-3,3,3′,3′-tetramethylindotricarbocyanine iodide; DiIC18 (7)]–labeled EVs and radioisotope-labeled EV signals are monitored clearly and tend to accumulate in the liver and spleen (102).

More recent genetic approaches to the study of EV release and uptake have taken advantage of fusion proteins engineered between EV tetraspanins and fluorescent reporters like green fluorescent protein (GFP), tdTomato, and mCherry (103, 104). While there are some limitations, the ability to follow EV biogenesis with genetic tools is a powerful technique for *in vitro* and *in vivo* models. It is important to note that, while the expression of tagged tetraspanins have some limitations (105), recent reports show that luminescent tagging of CD63-Nluc can be used to their distribution to the lung and spleen (102). Using a GFP tag, CD63-EGFP–labeled EVs can be detected in the parenchyma of the liver and spleen after intravenous injection (105). *In vivo* EV labeling via transgenic inducible GFP EV reporter mouse that contain a Cre recombinase–inducible promoter-driven CD9 fused to GFP provides the insight for EV distribution that depends on cellular source (106). With the use of tissue-specific expression of Cre recombinase, transgenic mice can produce CD81-mNG EVs. These EV can then be used for mapping cell-type–specific contribution to blood exosome population (107). These studies show that fluorescent and luminescent reporters fused with tetraspanins are useful for tracking EV tropism.

EVs of different sizes have their own N-glycosylation, protein, lipid, and nucleic acid profiles and show different biodistribution patterns. At 24 h after intravenous injection, a wide range of EV sizes were taken up by various organs (84% by liver, 14% by spleen, and 1.6% by bone marrow). Although almost all EVs were taken up in the liver, large EVs displayed lymph node tropism (108). Previous reports have summarized the biodistribution of EVs (109), with many small EVs being cleared from the blood within 1 h. A recent study has examined EVs from multiple cell types and assessed their biodistribution. In this example, EVs derived from B16BL6 melanoma, C2C12 myoblast, NIH3T3 fibroblast, MAEC aortic endothelial cell, or RAW264.7 macrophage were administered via intravenous injection. This study showed efficient clearance from the circulation into the liver (110). These reports help understand the biodistribution of EVs *in vivo* and *in vitro* and help guide the design and testing of future engineered EVs (**Figure 2B**).

mNG (mNeon Green)–labeled HEK293 cell–derived EVs intravenously injected accumulate in hepatocytes. Differential expression analysis of recipient cells revealed that cells dosed with a low number of EVs showed gene expression involved in EV biogenesis, endocytosis, and homeostasis. In cells dosed with a high number of EV, expressed genes related to lysosome acidification, assembly, and localization (29). HEK293 cell lines are cells commonly used for transfection and are derived from human kidney. EVs derived from the human embryonic kidney cell line HEK293 accumulate in the liver and are used for liver diseases (111, 112). EVs derived from HEK 293 cells expressing CD9 and CD63 show a similar biodistribution trend toward the liver and spleen. In addition, the relative distribution of CD63-positive EVs was enhanced in the gastrointestinal tract and reduced in the lung (113).

MSC-derived EVs show injury-related organ tropism. MSC EVs show a homing effect in the lung and liver via integrin-mediated protein interaction (114, 115). In addition, MSC-EVs accumulate in ischemic kidney and proximal tubules and affect renal tubule repair (116). Placental EVs associate with lung interstitial macrophage and liver Kupffer cells in an integrin-mediated manner. Placental EVs target maternal immune cells and cause genetic alteration of maternal immune cells (**Figure 2C**) (117).

Cancer-derived EVs recognize early-stage neoplastic tissues with tumor-tropism mimicking the membrane structures of cancer cells and promote their uptake into tumor (118, 119). Tumor tropic cancer–derived EVs occur without specificity for donor cancer types (120). Cancer cells involved in metastasis produce EVs with tropism for the relevant organ. Lung metastatic hepatocellular carcinoma exhibits lung tropism. Solute carrier organic anion transporter family member 2A1, alanine aminopeptidase, and chloride intracellular channel 1 can be found to be involved in lung-tropic proteins of EVs (121, 122). These reports suggest organ distribution according to donor cell type, and most show tropism to the liver, spleen, lung, and kidney. Using this cell- or organ-tropism information, it may be possible to target delivery of EV cargos through direct or indirect strategies that take advantage of molecules that can be displayed on EVs to mediate tropism in the appropriate microenvironment (**Figure 2D**).

## Tropism based on intracellular localization

Cell-to-cell communication by EVs through cargo delivery begins with EV uptake by recipient cells (123, 124). EV uptake is mediated by various mechanisms that likely depend on physical properties such as size and protein and lipid composition of the membrane (125–127). Clathrin-mediated endocytosis and micropinocytosis are considered a main route of EV uptake through a specific pathway but not necessarily through a specific receptor. In this pathway, uptake occurs as early as 15 min after introduction in an energy-dependent manner (128–131). Additionally, depending on the characteristics of EVs, pathways such lipid raft-mediated endocytosis have also been reported (132–134). EVs also indirectly deliver their cargos by fusion with plasma membrane of recipient cells (32, 131, 135). Internalization of EVs is captured by integrin β_3_–interacting HSPGs. This internalization process is regulated by the integrin β_3_–focal adhesion kinase (FAK), which is activated by EVs in an integrin β_3_–dependent manner and required for endocytosis (136).

Endosomal escape of EVs in the recipient cells is an important intracellular step in the delivery of EV cargo necessary to complete their function. Internalized EVs form intracellular vesicles that fuse with endosomes to form early endosomes. These early endosomes contain EVs that often fuse with lysosomes followed by endo-lysosome acidification (137). Overall, EV cargos are thought to be rapidly digested by autophagy in recipient cells (32, 138). Using CD63-pHluo (pH-sensitive fluorescent)–labeled EV, acidification of endocytosed EVs occurred in a narrower time range, from 2 min to 11 min (139). However, internalized EVs that undergo endosomal escape, these EVs can deliver functional cargos into recipient cells (140). EV cargo exposure and/or release to the cytosol occurs when endosomes are acidified by fusion with lysosomes (129, 141). In this step, endosomal acidification is involved in the fusion of EV and endosomal membrane followed by cargo release (32, 129). Chloroquine, which improves endosomal acidification, results in unstable endosomes and increased release of EV cargo into the cytoplasm. Bafilomycin A inhibits the proton transfer of vacuolar type H+-ATPase (V-ATPase) resulting in accumulation of endosomal cargo and inhibition of releasing EV cargos (142). Advances in engineering approaches and drugs should lead to better efficiencies of endosomal escape of macromolecules, such as proteins, nucleic acids, and lipid nanoparticles (143–145). Recent examples include ligands such as glycans (146), fusogenic proteins (147–149), and endocytosis related proteins (150) that can enhance endosomal escape in recipient cells (151). A study was also reported to improve target delivery efficiency by depleting integrin β_1_ to avoid non-specific clearance (152).

Advances in the tracking EV release from cells have been largely guided by technology. In early EV studies, electron microscopy, along with immunogold staining, was the standard for high-resolution imaging (153, 154). More recently, genetic reporters such as GFP or luciferase have been expressed as fusion proteins with classic tetraspanins (155). These studies have greatly advanced the ability to identify which cells express EVs and, in some studies, assess the uptake of those EVs where advanced high-resolution imaging is used because single EVs are smaller than the resolution of standard light microscopy (156). In another example of advances in technology, to identify the release of EVs upon release, pHlourin reporters have been used because they are pH-dependent ratiometric reporters. With this kind of reporter, as an EV is trafficked to the neutral extracellular space, fluorescence is increased upon neutralization and thereby tracked in various *in vitro* models (139) and *in vivo* (156) models. We and others have recently shown how cell-type–specific promoters can be used to direct the expression of such fusion reporters (i.e., CD9-GFP) in specific cell types in mice (106, 107, 157). As these tools develop more sophisticated reporters and cell-type–specific promoters and access more advanced imaging increases, we anticipate that the visualization of engineered EVs will become more useful and prevalent.

## Regulation of immune microenvironment by EV tropism

EVs are known to regulate cell-to-cell communication in the microenvironment of injury and cancer, with immune cells having a major role in modulation of the inflammation responses associated with wound repair and blockade of tumor progression. Here, we focus on examples of tissue injury where macrophage-derived EVs regulate fibroblasts in the wound bed (157) and on changes in EVs draining in mesenteric lymph–regulating immune responses in sentinel lymph nodes (158, 159) and tumor microenvironments. In cancer, one of the earliest observations was that integrins had a major role in conditioning the metastatic niche (37). Additional examples included studies on the role of EVs derived from triple-negative breast cancer cell bearing CSF-1 that accelerated differentiation of monocyte to pro-inflammatory macrophages (160). Plant-derived EVs likely also have interesting but poorly defined surface proteins that were shown to promote the reprogramming of tumor-TAMs to induce the anti-tumorigenic infiltration of CD8^+^ T cells (161). The tropism of TAM-derived EVs can also be modified by display cyclic RGD peptides to deliver payloads that attenuate PD-L1 expression and enhance the activation of CD8^+^ T cells (162). Myeloid-derived suppressor cells (MDSCs) were also important regulators of the “cold” tumor microenvironment based on studies showing that MDSC-derived EVs dysregulated Wnt/β-catenin signaling pathway and decreased anti-tumoral responses (163).

**Figure 3:**
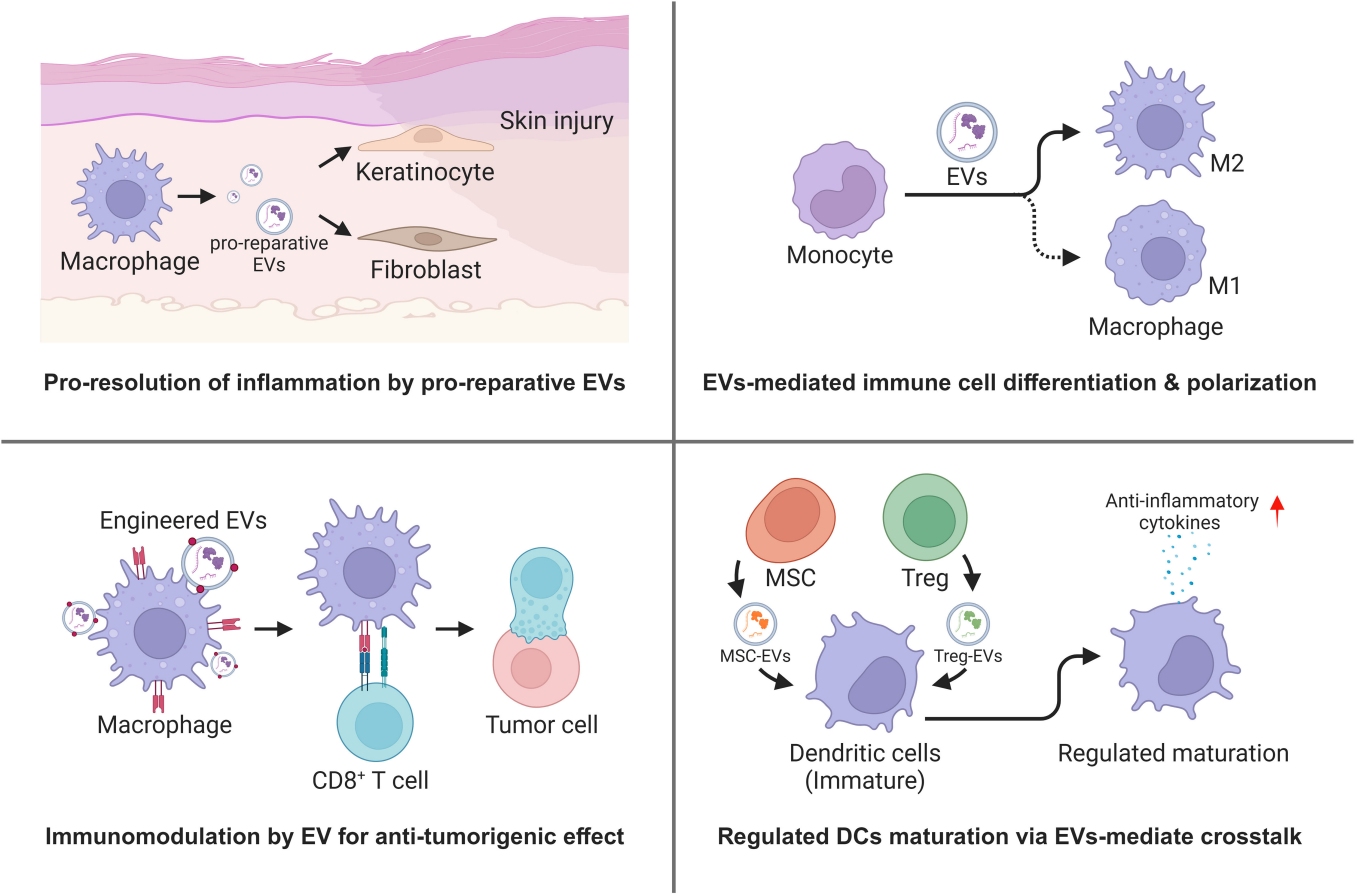
Regulation of immune microenvironment by EVs mediated immune cell crosstalk (Created with BioRender.com).

Among the most well-defined immune cell crosstalk responses in immunology is the interaction between regulatory T cells (Treg), CD8+ cytotoxic T cells, and DCs. Therefore, as the EV field develops within the field of adaptive immunity, consideration of EV tropism, EV cell source, and EV uptake will need to be addressed in more detail. Interesting interactions include EV-mediated transfer of miRNA contents that regulate IL-10 and IL-6 production (164) and MSC-derived EVs suppressing antigen uptake by immature DCs (165). EV tropism may also influence dysfunctional immune responses based on tissue-specific microenvironments. Therefore, engineered EVs that reverse these responses may yield pro-reparative, anti-inflammatory, and anti-tumorigenic immune responses depending on the disease state (**Figure 3**).

## Conclusions

EVs are key players in cell-to-cell communication, mediating various physiological signals in the body. Recent advances in the field have focused on understanding the biological activity of EVs and utilizing their internal cargo to apply EVs as therapeutics. However, a lack of understanding of tropism limits their clinical utility. Understanding the EVs tropism, from their surface composition by cell source and microenvironment to cellular uptake and systemic biodistribution, provides a pivotal junction between EV applications and clinical approaches. Depending on the EV source and recipient cells, there is an emerging literature that supports the translational potential of engineered EVs that consider the natural specificity of uptake pathways and surface display technologies.

## Author contributions

WC: Conceptualization, Investigation, Visualization, Writing – original draft, Writing – review & editing. DP: Writing – review & editing. BE: Conceptualization, Funding acquisition, Project administration, Supervision, Writing – review & editing.
